# Spatiotemporal Trends in Oral Cancer Mortality and Potential Risks Associated with Heavy Metal Content in Taiwan Soil

**DOI:** 10.3390/ijerph7113916

**Published:** 2010-11-05

**Authors:** Chi-Ting Chiang, Ie-Bin Lian, Che-Chun Su, Kuo-Yang Tsai, Yu-Pin Lin, Tsun-Kuo Chang

**Affiliations:** 1 Department of Bioenvironmental Systems Engineering, National Taiwan University, No. 1, Section 4, Roosevelt Road, Taipei City 106, Taiwan; E-Mails: f92622003@ntu.edu.tw (C.-T.C.); yplin@ntu.edu.tw (Y.-P.L.); 2 Graduate Institute of Statistics & Information Science, National Changhua University of Education, No. 1, Jin-De Road, Changhua 500, Taiwan; E-Mail: maiblian@cc.ncue.edu.tw; 3 Department of Internal Medicine, Changhua Christian Hospital, No. 135, Nanxiao Street, Changhua 500, Taiwan; E-Mail: 115025@cch.org.tw; 4 Department of Dentistry, Changhua Christian Hospital, No. 135, Nanxiao Street, Changhua 500, Taiwan; E-Mail: 72837@cch.org.tw

**Keywords:** spatiotemporal, spatial autocorrelation, factor analysis, spatial regression, oral cancer, heavy metal, soil pollution

## Abstract

Central and Eastern Taiwan have alarmingly high oral cancer (OC) mortality rates, however, the effect of lifestyle factors such as betel chewing cannot fully explain the observed high-risk. Elevated concentrations of heavy metals in the soil reflect somewhat the levels of exposure to the human body, which may promote cancer development in local residents. This study assesses the space-time distribution of OC mortality in Taiwan, and its association with prime factors leading to soil heavy metal content. The current research obtained OC mortality data from the Atlas of Cancer Mortality in Taiwan, 1972–2001, and derived soil heavy metals content data from a nationwide survey carried out by ROCEPA in 1985. The exploratory data analyses showed that OC mortality rates in both genders had high spatial autocorrelation (Moran’s ***I*** = 0.6716 and 0.6318 for males and females). Factor analyses revealed three common factors (CFs) representing the major pattern of soil pollution in Taiwan. The results for Spatial Lag Models (SLM) showed that CF1 (Cr, Cu, Ni, and Zn) was most spatially related to male OC mortality which implicates that some metals in CF1 might play as promoters in OC etiology.

## 1. Introduction

Heavy metals are extremely persistent in the environment and can cause adverse effects on human health. Research has classified many heavy metals, including arsenic (As), chromium (Cr[VI]) and nickel (Ni[II]), as human carcinogens [1,2]. Conceptually, soil and the human body intake environmental heavy metals absorbed in various ways. Heavy metal content in soil is an index of possible environmental exposure to heavy metal, and reflects somewhat the level of exposure of the human body. Some studies indicate that long-term exposure to heavy metals may promote cancer development in local residents [3–5]. Consulting the Taiwan Cancer Registry Database (TCRD) shows that oral cancer (OC) is more frequent in males, ranking as the fourth leading cause of cancer-related deaths in Taiwan since 2003. Several areas in the central and eastern parts of Taiwan, e.g., particularly Changhua County and Taitung County, display persistently high incidence rates of OC [6,7]. Betel quid chewing (BQC) and cigarette smoking (CS) are the established risks for OC in Taiwan [8,9].

Although the percentage of BQC prevalence in Taiwan has declined from 1996 to 2002, especially the maximum decrease from 18.9% to 9.3% in the middle area of Taiwan and the second-highest decrease from 44.0% to 36.6% in the eastern part of Taiwan [10], OC morbidity and mortality rates have been rising continually for several decades [11].

Industrialization and urbanization in Taiwan over the past two decades have chronically polluted a huge amount of farm soil due to the discharge of industrial wastewaters into the irrigation systems. In Taiwan, Changhua County has the highest Cr and Ni levels in farm soil [12,13]. Taitung County is located in Eastern Taiwan with low industrial pollution. The longitudinal Valley of Eastern Taiwan lies in the convergence zone between the Eurasia Plate and the Philippine Sea Plate. Volcanic activity accompanies an arc-continent collision that causes wide distribution of serpentine minerals in the coastal range of Eastern Taiwan [14]. Since serpentine soils generally contain very high Cr and Ni levels, some areas of Eastern Taiwan have relatively high levels of Cr and Ni [15].

Epidemiological studies have widely used spatial analyses in recent years to identify possible factors related to the occurrence of various diseases, including cancers and other health concerns [16]. Spatial clustering methods such as Moran-based statistics can identify the “hot spot” of a disease, which can be used to analyze the space-time structure of disease phenomenon clustered across space and time [17,18]. Additionally, spatial regression analysis quantifies the spatial pattern through creating a specific-contiguity weight and examining the relationship between the attributes of interest and latent explanatory variables that can interpret the observed spatial pattern [19].

Alcohol, particularly when associated with tobacco use, has been recognized as a critical risk factor for OC for nearly 50 years [20]; therefore, the most important etiological factors for OC are BQC, CS, and alcohol consumption activities [21–24]. Few studies have explored the potential effects of environmental factors as risks for OC. This study applied geographic information system (GIS) technology to map and visualize geographical clusters with significantly high factor scores and high OC mortality in Taiwan. This work focused on cartographic and geo-statistical methods in representing the geographical correlations among prime factors of soil pollution and OC mortality. Dimension reduction procedures are essential in any type of regression especially when the number of variables is relatively large. Among them, the principal component as well as factor analysis is considered to be efficient and very frequently used dimension reduction method, which had been incorporated in various spatial studies [25]. In this work, we integrated factor analysis results into the spatial lag model (SLM) regression to depict the temporal-spatial relationship between soil heavy metals and human mortality in OC and determined which heavy metal was the most critical environmental factor in OC etiology.

## 2. Materials and Methods

### 2.1. OC Mortality Rates

The current study obtained OC mortality from the Atlas of Cancer Mortality in Taiwan constructed in 2003, which contains OC age-standardized mortality rates (ASMR) in both genders of each township for each decade from 1972 to 2001.

### 2.2. Data on Soil Heavy Metal Content

Soil data were derived from a nationwide survey that determined the content in agricultural topsoil (0–15 cm) of arsenic (As), cadmium (Cd), chromium (Cr), copper (Cu), mercury (Hg), nickel (Ni), lead (Pb) and zinc (Zn) content, obtained from the Environmental Protection Administration (EPA) in Taiwan from 1983 to 1986 [12]. The total concentration of extractable As and Hg in the soil was determined by the aqua regia method, as well as the other six heavy metals by the 0.1 N HCl extraction method. A grid cell size of 1,600 ha was used as a sampling unit and 936 soil samples were collected across Taiwan. The area-weighted mean value represented the soil heavy metal content in each township [26]. A total of 231 townships have information on OC mortality rates in males and soil heavy metal content was used in the spatial regression analysis.

### 2.3. Factor Analysis

Factor analysis is a widely used multivariate statistical method for dimension reduction. The analysis recombines original variables into fewer underlying factors called common factors (CFs) that retain as much information from the original variables as possible. For large data sets, the CFs can effectively reduce information retrieval complexity. The eigenvalue quantifies the contribution of each CF to total variance. The factor loadings are in the range of −1 to 1, and the greater absolute value of the factor loading indicates a stronger relationship between the independent CFs and variables. Factor loadings are classified as strong, moderate, and weak corresponding to absolute loading values in the range of >0.75, 0.75–0.50 and 0.50–0.30, proposed by Liu *et al.* [27]; therefore, in this study, factor loadings greater than 0.75 (strong) in absolute value were used to make decisions regarding significant loading. The factor scores were computed for each case, *i.e.* township by regression method to express the contribution of each CF to each case. This study performed factor analysis to determine the major factors influencing the soil pollution pattern in Taiwan.

### 2.4. Moran-based Autocorrelation Statistics

Spatial autocorrelation exists when a value observed in one location depends on the values at neighboring locations. The Moran Index (Moran’s ***I***) is a global spatial autocorrelation statistic used to quantify the degree of spatial similarity among neighboring observations over the study area. The space-time Moran’s ***I*** (STI) is an extension of Moran’s ***I***, originally proposed by Wartenberg [28]. The STI computes the relationship between the spatial lag at time t and the original variable at time *t-k* (*k* is the order of the time lag). Therefore, STI quantifies the effect that a change in a spatial variable, operated in the past (*t-k*) in an individual location *i* exerts over its neighborhood at present. The local Moran Index (Moran’s ***I****_i_*), which decomposes the global Moran’s ***I*** statistic into contributions for each location, is termed local indicators of spatial association (LISA) [29]. Specifically, the global Moran’s ***I*** is a weighted average of local Moran’s ***I****_i_*. The null hypothesis of the spatial autocorrelation test is that the OC mortality is not associated with neighboring township levels, *i.e.*, there is no spatial autocorrelation. The alternate hypothesis is that spatial clustering exists, *i.e.*, neighboring townships have a similar OC mortality.

### 2.5. Spatial Regression Analysis

Studies commonly use multivariate linear regression analyses to determine the relationships between environmental factors and diseases, including cancers [30,31]. However, for the spatial data, the fundamental assumptions of the classical linear model (seen in matrix form, **y** = **X**β + ɛ, where **y** is an *n* × 1 vector of observation on the dependent variable, **X** is an *n* × 1 vector of observation on the explanatory variable, β is an regression coefficient for the explanatory variable, and ɛ is an *n* × 1 vector of random error term) are violated, due to the spatial autocorrelation among regression residuals. The SLM is a spatial regression method, which can incorporate spatial dependency into the classical regression model. The SLM adds an additional predictor in the form of a spatially lagged exogenous variable, with a formula expressed as **y** = ρ**Wy** + **X**β + ɛ, where ρ is a spatial autoregressive coefficient of the spatial lag term, **W** is an *n* × *n* binary matrix of spatial weights, **Wy** is the spatially lagged dependent variable, and the other notation is as before. The maximum likelihood method estimates the SLM parameters.

This study constructs a contiguity-based spatial weight for each township by queen contiguity relationships, which defines spatial neighbors as areas with a shared border and vertexes [32]. In order to alleviate the effect due to unequal number of neighbors, a conventional row-standardization of the original spatial weight matrix **W** was used with neighborhoods based on polygon contiguity. Let **W** with elements *w̃_ij_* be a spatial neighbor matrix, and each element *w̃_ij_* was divided by the sum of the elements in the row. Note the (*i*, *j*)th element of a spatial weight matrix **W**, denoted *w̃_ij_*, quantifies the spatial dependent between locations *i* and *j*. A row-standardized weight **W̃** means that each row of the weight matrix must sum to one, which is defined by **W̃** = *w̃_ij_/*∑*_j_w̃_ij_*. The hypothesis regarding exploratory spatial analyses of OC mortality in Taiwan was tested using a free software program called the GeoDa version 0.9.5-I, developed by Luc Anselin [33]. For a statistical inference, 999 Monte Carlo permutations were performed with the significance level set as 0.05.

## 3. Results and Discussion

### 3.1. Spatial and Space-time Autocorrelation of OC Mortality

Table 1 shows the positive and statistically significant spatial autocorrelations for male and female OC mortality rates in the three 10-year periods. All of the STI values exhibit significant high time-lagged values, showing increasing trends in OC mortality rates. The influence of past OC mortality rates in a certain location over its neighborhood in the present increases with time. However, note that a limitation of the contiguity-based method of defining spatial weights cannot reflect real “neighborhood” due to ignorance of the topographical effects.

### 3.2. Factor Analysis for Heavy Metals in Soil

The factor analysis generated three CFs with eigenvalues greater than one, which retain 73.78% of the total variance. Table 2 gives the resulting factor loadings, eigenvalues, and cumulative percentage of variance of each of the three CFs after rotation.

This study considers a variable (metal) with factor loadings greater than 0.75 to be important in the CF. Using this criterion, the first common factor (CF1) explained 42.02% of total variance with strong positive loadings on heavy metals Cr, Cu, Ni, and Zn. The association of Cr, Cu, Ni, and Zn in CF1 reflects the maximum influence of electroplating and other metal treatment plants on soil pollution in Taiwan, according to the results of previous research [34]. The second common factor (CF2) included heavy metals Cd and Pb, which explained 17.55% of the total variance. The association of Cd and Pb in CF2 reflects the influence of pigments and plastic factories on soil pollution. The third common factor (CF3) included As, which explained 14.21% of the total variance. However, the As content in soil closely relates to parent materials.

### 3.3. Spatial Clusters of OC Mortality and Related Common Factors

**Figure 1:**
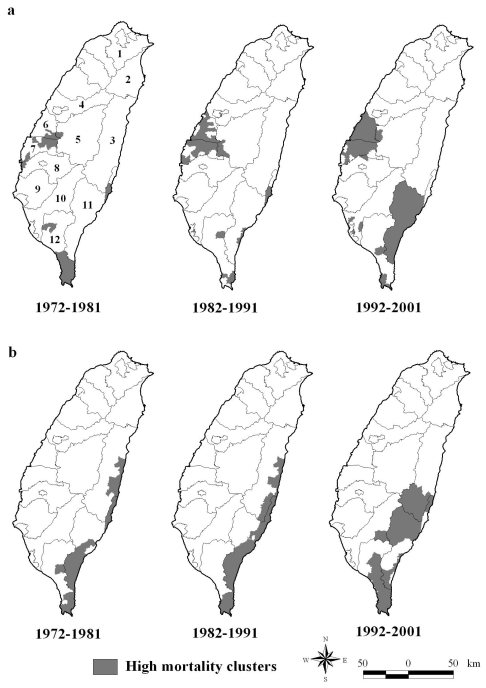
Statistically significant high-mortality clusters of OC, 1972–2001. a. Male and b. Female. (1; Taipei County), (2; Yilan County), (3; Hualien County), (4; Taichung County), (5; Nantou County), (6; Changhua County), (7; Yunlin County), (8; Chiayi County), (9; Tainan County), (10; Kaohsiung County), (11; Taitung County), (12; Pingtung County).

Figure 1 displays a map showing the geographic distribution of high OC mortality rates for both genders in the three 10-year periods from 1972 to 2001. A few aggregation areas of high OC mortality were observed and mainly scattered in the central and southernmost part of Taiwan during 1972–1981. Over the period 1982 to 1991, a single high- mortality cluster of male OC, located in the central region of Taiwan, centered on Changhua and Yunlin Counties; however, the previous cluster in southernmost Taiwan disappeared. From 1992 to 2001, the map identified two apparently large-scale clusters of high male OC mortality. In addition to the previous cluster in Central Taiwan, the other exhibited in Taitung County of Eastern Taiwan. The location of high-mortality clusters of male OC in Central Taiwan gradually expended to include the entire Changhua County during the period from 1972 to 2001. The main aggregation areas of high OC mortality for females emerged in Taitung and Hualien Counties of Eastern Taiwan over the past thirty years.

**Figure 2:**
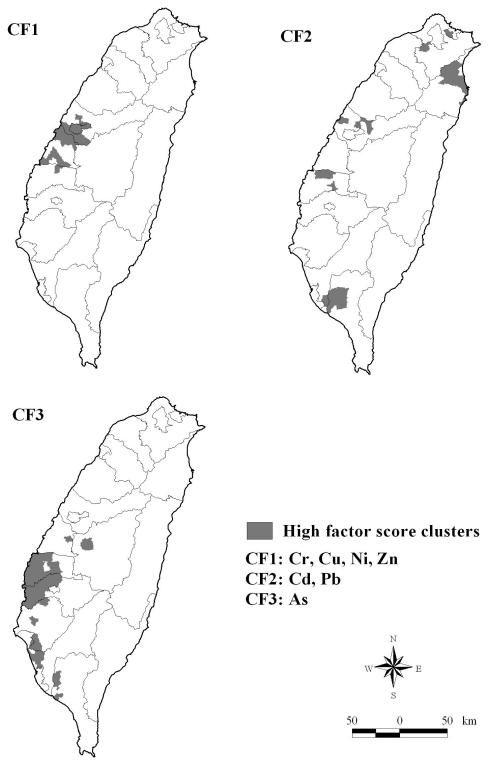
Statistically significant high factor score clusters.

Figure 2 shows the spatial clusters of high CF1 scores mainly in the adjacent area of Taichung and Changhua Counties in Central Taiwan. Yilan and Pingtung Counties show two distant and small-area cluster distributions of high CF2 scores. Yunlin and Chiayi Counties show a major cluster of high CF3 scores.

In the early 1970’s, Central and Southern Taiwan showed a scattered distribution of high-mortality spots for male OC. The spots not only expanded with time, but also clustered. During 1992–2001, the high-mortality region of male OC covered the entire Changhua County. Meanwhile, the other significant high-mortality cluster of male OC exhibited in Taitung County. Since the 1970s, Changhua County has become an aggregation of electroplating and hardware manufacturing factories due to the government policy to promote “homes into small factories.” Changhua County is a well-known Taiwanese “rice warehouse,” and more than 60% of the county’s total area has become arable land since 2001 [35]. Wastewater discharged from factories into the cultivated farmland during the past few decades has caused heavy metal pollution of farm soil in Changhua [36]. A positive (STL > 0) time-trend exists in spatial dependence of OC mortality. These results show that the temporal changes in spatial clusters of OC mortality may relate to environmental pollution. However, high-mortality clusters of female OC located in Eastern Taiwan at any time period are mainly because BQC has become a unique part of the culture of Taiwanese aborigines for some time, regardless of their gender. The CF1 obtained from factor analysis mainly represented four metals: Ni, Cr, Zn, and Cu. The spatial clusters of high CF1 scores were located mainly in the central part of Taiwan, especially in Changhua County. The spatial locations of clusters with the high CF3 scores closely relate to the spatial distribution pattern of As content in soil parent materials [37]. As a result, the geographical distribution of high soil as content in areas of Southwestern Taiwan coincides with high CF3 scores.

### 3.4. Spatial Lag Models (SLM) for Male OC Mortality Rates

This study further explored whether there is any relationship between the abnormally high-mortality rates of male OC and environmental risk factors, *i.e.*, CF1, CF2, and CF3. The Moran’s ***I*** statistic showed the spatial autocorrelation in residuals for ordinary least squares (OLS) regression, as well as both Lagrange multipliers (LM), and Robust LM for spatial lag were statistically significant in favor of conducting SLM regression. The current research used three CFs as explanatory variables to perform SLM regression. Table 3 shows the estimation results of SLM regressions for male OC mortality rates in the three 10-year periods from 1972 to 2001. The CF1 significantly and positively associated with male OC mortality rates in the three SLM regressions for the three 10-year periods. The significant regression coefficients (β) for CF1 were 0.296, 0.402, and 0.497 and the percentages of variance explained (R^2^) were 0.304, 0.362, and 0.533; therefore, the above results implied a positive spatial correlation between CF1 and male OC mortality, that is, the magnitude of male OC mortality in Taiwan may be associated with the contents of heavy metals Cr, Cu, Ni, and Zn, the major components of CF1. In 1992–2001, CF3 also associated with male OC mortality (β = 0.605). Additionally, the estimation of all three models generated significantly positive values (ρ > 0) for spatial effect.

The spatial regression results showed that only CF1 positively associated with male OC mortality in all three-time periods, indicating that higher CF1 scores in areas had higher male OC mortality rates. Chromium and Ni are widely used industrial chemicals, and sufficient evidence exists that Cr and Ni compounds pose a carcinogenic risk to humans. Previous investigations revealed that the whole blood (B-Cr) and urinary Ni (U-Ni) levels of Cr of local residents living in the factory-dense areas of Changhua County were higher than those in other areas [39,40].

High levels of Cr and Ni in soil result from Cr- and Ni-emitting industrial sources in Changhua County, Central Taiwan [36]. However, weathered serpentine parent materials cause very high levels of Cr and Ni in the soil of Eastern Taiwan, ranging from 400 mg kg^−1^ to 3,300 mg kg^−1^ of Cr and 400 mg kg^−1^ to 5,800 mg kg^−1^ of Ni [15,41]. A recent study revealed that maximum concentrations of Cr and Ni in the grains of brown rice grown on serpentine soils in Eastern Taiwan were 4.48 mg kg^−1^ dry wt. and 6.71 mg kg^−1^ dry wt. [42], apparently higher than the normal Cr and Ni average values in Taiwan’s brown rice of 0.14 kg^−1^dry wt. and 0.47 mg kg^−1^dry wt. [43]. Nevertheless, the bioavailability and mobility of Ni in soils were much higher than those of Cr [14]. This result shows that agricultural crops grown in soils with high Ni content may accumulate considerable amounts of Ni in their tissues. However, cancers are chronic diseases, whose developments often require long-term human exposure to environmental risk factors; therefore, it is necessary to address the fact that limitations of human migration and movement may make the locations of human exposure to heavy metals and OC occurrence likely to be not exactly the same. Further investigation is needed on the link between this food chain and potential health hazards for humans.

## 4. Conclusions

This study assessed the association between soil heavy metal content and OC mortality. Certain heavy metals are well-known to be human carcinogens. Various experimental and epidemiological studies in human populations exposed to these carcinogenic heavy metals via the environment provide evidence of a causal link to specific human cancers. Chromium and Ni are ubiquitous environmental and industrial contaminants. Chromium was found to be a potent inducer of oral-cavity tumor growth in experimental animals, and neoplastically transformed cells in culture [44,45]. Nickel can cause cell transformation and induce tumors in animal models [46]. This study concludes that areas having high Ni or Cr content in the soil, from sources involving either anthropogenic or non-anthropogenic pollution, spatially correlates with regions of high male OC mortality in Taiwan, and provides direction for further investigations to verify the role of heavy metal Ni or Cr in the development and progression of OC.

## Figures and Tables

**Table 1:** Global Moran’s ***I*** and space-time Moran’s ***I*** (STI) of OC mortality rates, 1972–2001.

Periods	Male		Female	
	Moran’s *I*	STI	Moran’s *I*	STI
1972–1981	*0.3659		*0.2700	
1982–1991	*0.4102	*0.3146	*0.4470	*0.2875
1992–2001	*0.6013	*0.5108	*0.4553	*0.3957
1972–2001	*0.6716		*0.6318	

* p < 0.05.

**Table 2:** Results from the factor analysis for heavy metals in soil.

Variable	CF1	CF2	CF3
As	−0.06	0.00	**0.95**
Cd	0.02	**0.85**	0.16
Cr	**0.86**	0.12	−0.13
Cu	**0.83**	0.09	−0.30
Hg	0.54	0.14	0.20
Ni	**0.88**	0.00	0.09
Pb	0.25	**0.78**	−0.19
Zn	**0.91**	0.19	−0.14

Eigenvalue	3.36	1.40	1.14
% Total variance	42.02	17.55	14.21
Cumulative % variance	42.02	59.56	73.78^a^

a Total cumulative variance. The loadings whose absolute value is greater than 0.75 of the total variance were in bold.

**Table 3:** Estimations of spatial lag models (SLM) for male OC mortality rates.

OC mortality (Y)	Variables (X)^a^	β^b^	ρ^c^	R^2^^d^
1972–1981	CF1	*0.296	**0.500	0.304
	CF2	0.069		
	CF3	0.253		

1982–1991	CF1	**0.402	**0.565	0.362
	CF2	0.034		
	CF3	0.207		

1992–2001	CF1	*0.497	**0.651	0.533
	CF2	0.031		
	CF3	*0.605		

a Explanatory variables of three common factors included CF1, CF2 and CF3 were obtained by factor analysis applied to eight heavy metals data.

b β expresses the regression coefficients.

c ρ expresses the spatial autoregressive coefficients.

d R^2^ (the percentage of variation explained) is not directly provided for spatial model, and model fit is thus assessed with a pseudo-R^2^ value calculated as the squared Pearson correlation between predicted and observed values [38].

* p < 0.05 and

** p < 0.01.

## References

[1] IARC (International Agency for Research on Cancer) Overall Evaluations of Carcinogenicity: An Updating of IARC Monographs 1–42 IARC Lyon, France 1987

[2] IARC (International Agency for Research on Cancer) Chromium, Nickel and Welding IARC Lyon, France 1990

[3] Rheeder J Marasas WFO Farina MPW Thompson GR Nelson PE Soil fertility factors in relation to esophageal cancer risk areas in Transkei, southern Africa Eur. J. Cancer Prev 1994 3 49 56 8130716 10.1097/00008469-199401000-00007

[4] Türkdoğan MK Kilicel F Kara K Tuncer I Uygan I Heavy metals in soil, vegetables and fruits in the endemic upper gastrointestinal cancer region of Turkey Environ. Toxicol. Pharmacol 2002 13 175 179 10.1016/S1382-6689(02)00156-4 21782652

[5] Lalor GC Review of cadmium transfers from soil to humans and its health effects and Jamaican environment Sci. Total. Environ 2008 400 162 172 18752835 10.1016/j.scitotenv.2008.07.011

[6] Chiang CT Hwang YH Su CC Tsai KY Lian IB Yuan TH Chang TK Elucidating the underlying causes of oral cancer through spatial clustering in high-risk areas of Taiwan with a distinct gender ratio of incidence Geospatial Health 2010 4 230 242 20503191 10.4081/gh.2010.203

[7] Su CC Chung JA Hsu YY Huang SJ Lian IB Age at diagnosis and prognosis of oral cancer in relation to the patient’s residential area: experience from a medical center in Taiwan Oral Oncol 2008 44 1032 1038 18620898 10.1016/j.oraloncology.2008.01.015

[8] Lu CT Yen YY Ho CS Ko YC Tsai CC Hsieh CC Lan SJ A case-control study of oral cancer in Changhua County, Taiwan J. Oral Pathol. Med 1996 25 245 248 8835822 10.1111/j.1600-0714.1996.tb01379.x

[9] Wen CP Tsai SP Cheng TY Chen CJ Levy DT Yang HJ Eriksen MP Uncovering the relation between betel quid chewing and cigarette smoking in Taiwan Tob. Control 2005 14 Supplement 1 i16 i22 15923442 10.1136/tc.2004.008003 PMC1766184

[10] Yang YH Chen HR Tseng CH Shieh TY Prevalence rates of areca/betel quid chewing in counties of Taiwan (in Chinese) Taiwan J. Oral Med. Health Sci 2002 18 1 16

[11] Su CC Yang HF Huang SJ Lian IB Distinctive features of oral cancer in Changhua County: high incidence, buccal mucosa preponderance, and a close relation to betel quid chewing habit J. Formos. Med. Assoc 2007 106 225 233 17389167 10.1016/s0929-6646(09)60244-8

[12] ROCEPA (Environmental Protection Administration of the Republic of China) Survey of Heavy Metals in the Soil Samples Yearbook of Environmental Protection Statistics Taiwan Area, the Republic of China ROEPA Taipei, Taiwan 1985

[13] Su CC Lin YY Chang TK Chiang CT Chung JA Hsu YY Lian IB Incidence of oral cancer in relation to nickel and arsenic concentrations in farm soils of patients’ residential areas in Taiwan BMC Public Health 2010 10 67 20152030 10.1186/1471-2458-10-67 PMC2834627

[14] Hseu ZY Tsai H Hsi HC Chen YC Weathering sequences of clay minerals in soils along a serpentinitic toposequence Clay Clay Miner 2007 55 389 401

[15] Cheng CH Jien SH Tsai H Chang YH Chen YC Hseu ZY Geochemical element differentiation in serpentine soils from the ophiolite complexes, eastern Taiwan Soil Sci 2009 174 283 291

[16] Jerrett M Burnett RT Goldberg MS Sears M Krewski D Catalan R Kanaroglou P Giovis C Finkelstein N Spatial analysis for environmental health research: concepts, methods, and examples J. Toxicol. Environ. Health Part A 2003 66 1783 1810 12959844 10.1080/15287390306446

[17] Fang L Yan L Liang S de Vlas SJ Feng D Han X Zhao W Xu B Bian L Yang H Gong P Richardus JH Cao W Spatial analysis of hemorrhagic fever with renal syndrome in China BMC Infect. Dis 2006 6 77 16638156 10.1186/1471-2334-6-77 PMC1471792

[18] Rainey JJ Omenah D Sumba PO Moormann AM Rochford R Wilson ML Spatial clustering of endemic Burkitt’s lymphoma in high-risk regions of Kenya Int. J. Cancer 2006 120 121 127 17019706 10.1002/ijc.22179

[19] Zeng DD Yan O Li S Zeng D Chen HC Rolka H Lober B Waterman M Spatial regression-based environmental analysis in infectious disease informatics Biosurveillance and Biosecurity Springer-Verlag Berlin/Heidelberg, Germany 2008 175 181

[20] Ogden GR Alcohol and oral cancer Alcohol 2005 35 169 173 16054978 10.1016/j.alcohol.2005.04.002

[21] Blot WJ McLaughlin JK Winn DM Austin DF Greenberg RS Preston-Martin S Bernstein L Schoenberg JB Stemhagen A Fraumeni JF Jr Smoking and drinking in relation to oral and pharyngeal cancer Cancer Res 1988 48 3282 3287 3365707

[22] Choi SY Kahyo H Effect of cigarette smoking and alcohol consumption in the aetiology of cancer of the oral cavity, pharynx and larynx Int. J. Epidemiol 1991 20 878 885 1800426 10.1093/ije/20.4.878

[23] Merletti F Boffetta P Ciccone G Mashberg A Terracini B Role of tobacco and alcoholic beverages in the etiology of cancer of the oral cavity oropharynx in Torino, Italy Cancer Res 1989 49 4919 4924 2758421

[24] Cancela MD Ramadas K Fayette JM Thomas G Muwonge R Chapuis F Thara S Sankaranarayanan R Sauvaget C Alcohol intake and oral cavity cancer risk among men in a prospective study in Kerala, India Community Dent. Oral Epidemiol 2009 37 342 349 19486349 10.1111/j.1600-0528.2009.00475.x

[25] Novembre J Stephens M Interpreting principal component analyses of spatial population genetic variation Nature Genetics 2008 40 646 649 18425127 10.1038/ng.139 PMC3989108

[26] Davidson EA Spatial covariation of soil organic carbon, clay content, and drainage class at a regional scale Landscape Ecol 1995 10 349 362

[27] Liu CW Lin KH Kuo YM Application of factor analysis in the assessment of groundwater quality in a blackfoot disease area in Taiwan Sci. Total Environ 2003 313 77 89 12922062 10.1016/S0048-9697(02)00683-6

[28] Wartenberg D Multivariate spatial correlation—a method for exploratory geographical analysis Geogr. Anal 1985 17 263 283

[29] Anselin L Local indicators of spatial association–LISA Geogr. Anal 1995 27 93 115

[30] Ashley DJ Environmental factors in the aetiology of gastric cancer Br. J. Prev. Soc. Med 1969 23 187 189 5798461 10.1136/jech.23.3.187 PMC1059194

[31] Dyomin SN Buldacov LA Ternovsky IA Tokarskaya ZB Fomina TP Tretyakov FD Ivanova GN Shevchenko VN Kolmogortsev VA Uralshin AG Factors effecting the morbidity in populations living in the vicinity of atomic industry plants Sci. Total Environ 1994 142 105 109 8178128 10.1016/0048-9697(94)90078-7

[32] Lai PC So FM Chan KW Areal methods of disease analysis Spatial Epidemiological Approaches in Disease Mapping and Analysis CRC Press Boca Raton, FL, USA 2009 79

[33] Anselin L An Introduction to Spatial Autocorrelation Analysis with GeoData Spatial Analysis Laboratory University of Illinois Available online: http://geodacenter.asu.edu/learning/tutorials (accessed on 20 August 2010)

[34] Chang TK Hwang KT Shyu GS Using Factor Analysis to Evaluate Characteristic of Metals in Soil Pollution (in Chinese) J. Taiwan Agric. Eng 1997 43 11 18

[35] COA (Council of Agriculture) A gricultural Statistics Yearbook 2001 Council of Agriculture, Executive Yuan, R.O.C Taipei, Taiwan 2001

[36] Lin YP Teng TP Chang TK Multivariate analysis of soil heavy metal pollution and landscape pattern in Changhua county in Taiwan Landscape Urban Plan 2002 62 19 35

[37] Chang TK Shyu GS Lin YP Chang NC Geostatistical, analysis of soil arsenic content in Taiwan J. Environ. Sci. Health Part A 1999 34 1485 1501

[38] Kissling WD Carl G Spatial autocorrelation and the selection of simultaneous autoregressive models Global Ecol. Biogeogr 2008 17 59 71

[39] Chang FH Wang SL Huang YL Tsai MH Yu ST Chang LW Biomonitoring of chromium for residents of areas with a high density of electroplating factories J. Expo. Sci. Environ. Epidemiol 2006 16 138 146 16106258 10.1038/sj.jea.7500445

[40] Chang FH Wang HJ Wang SL Wang YC Hsieh DPH Chang LW Ko YC Survey of urinary nickel in residents of areas with a high density of electroplating factories Chemosphere 2006 65 1723 1730 16777179 10.1016/j.chemosphere.2006.04.083

[41] Hseu ZY Concentration and distribution of chromium and nickel fractions along a serpentinitic toposequence Soil Sci 2006 171 341 353

[42] Wu JH Soluble Heavy Metal and Rice Uptake from a Serpentine Soil with Different Fertilizer Treatments National Pingtung University of Science and Technology Pingtung, Taiwan 2009

[43] Lin HT Wong SS Li GC The concentrations of heavy metals in crops of Taiwan and the daily intake of heavy metals by R.O.C. people J. Chin. Agr. Chem. Soc 1992 30 463 470

[44] Stout MD Herbert RA Kissling GE Collins BJ Travlos GS Witt KL Melnick RL Abdo KM Malarkey DE Hooth MJ Hexavalent Chromium Is Carcinogenic to F344/N Rats and B6C3F1 Mice after Chronic Oral Exposure Environ. Health. Persp 2009 117 716 722 10.1289/ehp.0800208 PMC2685832 19479012

[45] Singh J McLean JA Pritchard DE Montaser A Patierno SR Sensitive quantitation of chromium-DNA adducts by industively coupled plasma mass spectrometry with a direct injection high-efficiency nebulizer Toxicol. Sci 1998 46 260 265 10048129 10.1006/toxs.1998.2512

[46] Kasprzak KS Sunderman FW Jr Salnikow K Nickel carcinogenesis Mutat. Res 2003 533 67 97 14643413 10.1016/j.mrfmmm.2003.08.021

